# Severe upper limb soft tissue infection with compartment syndrome following a scratch injury: a case report

**DOI:** 10.1186/s13256-026-06651-2

**Published:** 2026-09-30

**Authors:** Alexander Blümke, Suna Brunnemer, Frederik Weil, João Pinheiro, Frederic Bludau, Michael Hackl, Sascha Gravius, Ali Darwich

**Affiliations:** 1 Department of Orthopedics and Trauma Surgery, Universitätsklinikum Heidelberg – UK Mannheim GmbH, Medical Faculty Mannheim, Heidelberg University, Mannheim, Germany; 2 BG Klinik Ludwigshafen, Department of Trauma and Orthopedic Surgery, Heidelberg University, Ludwigshafen, Germany

**Keywords:** SSTI, Phlegmon, Compartment syndrome, Necrosectomy, Case report

## Abstract

**Background:**

Phlegmon is a severe skin and soft tissue infection (SSTI) that can escalate rapidly if not promptly recognized and treated. Its early presentation may appear deceptively mild, potentially delaying recognition of its severity. Without timely intervention, phlegmon can extend into deeper tissues and result in life-threatening systemic complications. Although SSTIs are common, progression from a seemingly minor scratch injury to a fulminant upper extremity infection complicated by compartment syndrome and multiorgan failure is uncommon. This case is reported to highlight the potential severity of such presentations and the importance of early recognition and intervention.

**Case presentation:**

We present the case of a 58-year-old German female teacher who developed a severe left upper extremity SSTI following a minor scratch injury sustained from a student. Despite initial conservative management with antibiotic therapy, the condition progressed to severe phlegmon with compartment syndrome, culminating in sepsis and multiorgan failure characterized by acute renal failure, disseminated intravascular coagulation (DIC), and early liver failure. Urgent surgical intervention was required, including fasciotomy, surgical debridement, and multiple revision procedures. Microbiological cultures initially identified *Streptococcus* *pyogenes* (Group A). During the later course of treatment, *Pseudomonas aeruginosa* was detected in revision samples. Empirical intravenous antibiotic therapy with Meropenem was initiated and subsequently adjusted to Ampicillin/Sulbactam according to microbiological findings. The patient required hemodialysis because of acute renal failure but gradually recovered, allowing discontinuation of dialysis. At follow-up, the infection had resolved and the skin grafts demonstrated satisfactory healing; however, significant functional deficits persisted despite intensive rehabilitation.

**Conclusion:**

This case underscores the critical importance of early diagnosis and aggressive multidisciplinary management in preventing severe complications associated with SSTIs. Furthermore, it highlights that seemingly minor skin injuries may progress to compartment syndrome and life-threatening systemic complications. Early recognition, prompt surgical intervention, and coordinated multidisciplinary care were essential for successful infection control and limb preservation.

## Introduction

Phlegmon, an acute and often rapidly progressing skin and soft tissue infection (SSTI), can result in severe complications if not diagnosed and treated promptly. Characterized by diffuse inflammation, phlegmon typically affects subcutaneous tissues and may lead to the formation of abscesses and necrosis if left untreated [1]. The condition is most commonly associated with bacterial infections, with *Streptococcus pyogenes* (Group A streptococcus) and *Staphylococcus aureus* being the predominant pathogens identified in clinical cases [2].

In recent years, the incidence of SSTIs has been increasing, particularly among patients with compromised immune systems or those with chronic underlying conditions [3]. Effective management of SSTIs involves a combination of prompt surgical intervention for debridement and appropriate antibiotic therapy. The choice of antibiotics may vary based on local resistance patterns; however, broad-spectrum agents are often employed in severe cases to cover a wide range of potential pathogens [4].

Despite advancements in medical and surgical therapies, deep-seated soft tissue infections, such as fulminant phlegmon and necrotizing fasciitis, frequently demonstrate rapid progression to sepsis, significantly elevating the risk of septic shock. Complications, including acute kidney injury requiring hemodialysis, are particularly common in severe cases with systemic involvement. Effective management of these patients demands a coordinated multidisciplinary approach involving infectious disease specialists, surgeons, and critical care teams to optimize clinical outcomes and minimize long-term sequelae.

This case report describes the unusual progression of a seemingly minor scratch injury to a fulminant upper extremity soft tissue infection caused by *Streptococcus pyogenes*, complicated by compartment syndrome, sepsis, disseminated intravascular coagulation (DIC), acute renal failure, and early liver failure. The case highlights the importance of early recognition and aggressive multidisciplinary management in preventing limb loss and further clinical deterioration.

## Case presentation

### Medical history

A 58-year-old German female patient was referred to our medical university center following a transfer from a local hospital. She had sustained a scratch injury to her left forearm approximately 2 weeks earlier (Day -14), when a student accidentally scratched her. Initially, the injury was managed conservatively with antibiotic administration; however, symptoms escalated to include pain, swelling, blister formation, and systemic signs of infection such as fever. She was admitted to the local hospital one day before her transfer (Day -1), where intravenous antibiotic therapy with Ampicillin/Sulbactam and Clindamycin was initiated. Due to the patient's worsening clinical condition, the referring hospital escalated the antibiotic therapy to Meropenem on the day of transfer (Day 0).

The patient’s prior medical history included a hypothyroidism treated with hormone replacement therapy, arterial hypertension, restless legs syndrome, and depression. One week prior to her admission, the patient reported having experienced a COVID-19 infection.

### Clinical examination

**Fig. 1:**
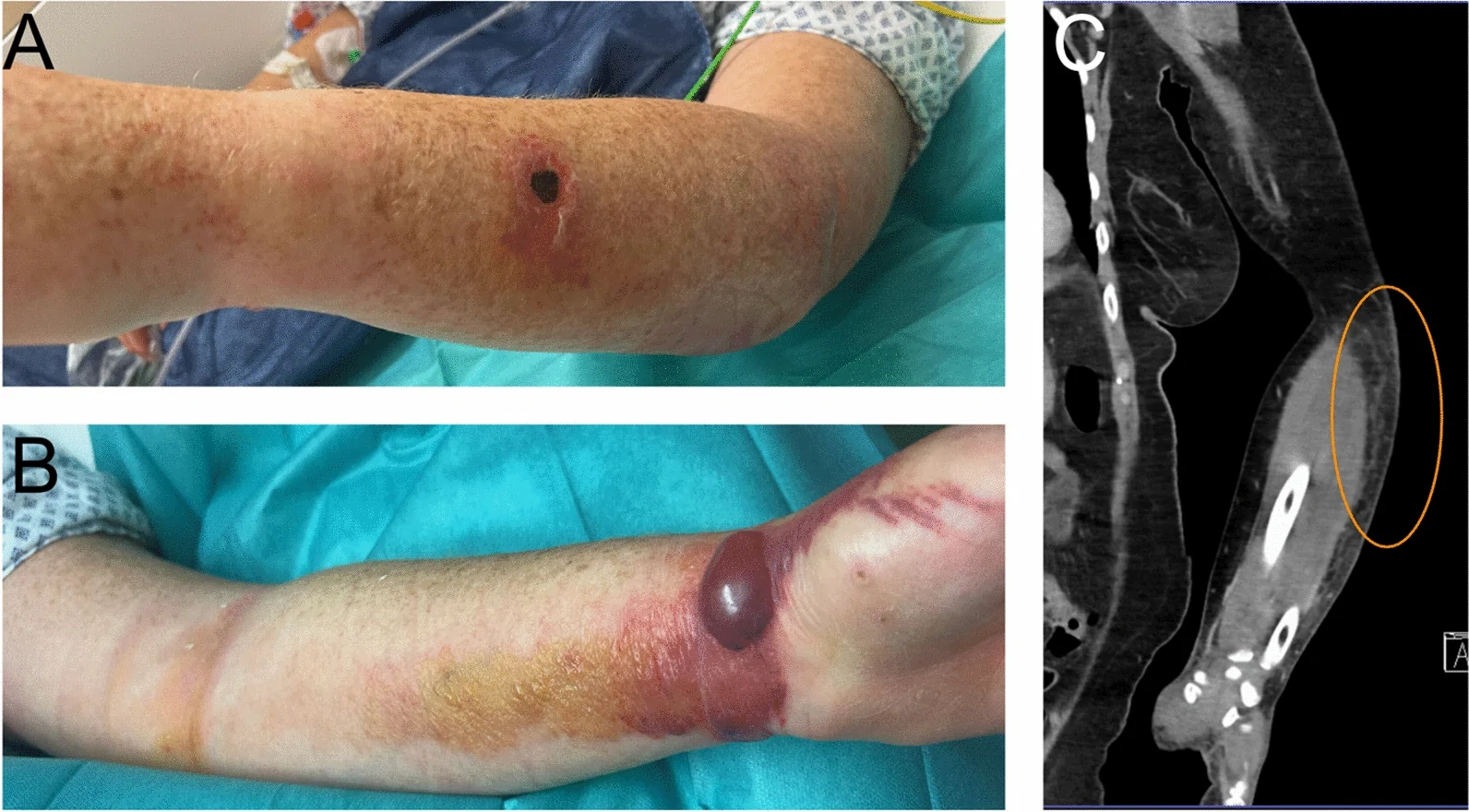
Clinical presentation at the time of admission. **a** The patient was initially scratched by a student on the dorsal side of the left forearm. **b** The left upper limb showed signs of blistering and necrosis. **c** Computed tomography images depict subcutaneous edema (orange circle), consistent with a soft tissue infection. There was no evidence of a local abscess or gas formation along the fascial planes. Furthermore, no signs of osteomyelitis were observed

Upon examination (Day 0), the patient presented with chills, along with a scratch wound on the dorsal side of the left forearm (Fig. 1a) and significant generalized swelling that was accompanied by blistering (Fig. 1b). A neurological examination revealed paresthesia in the median nerve distribution. Signs of compartment syndrome were evident, including severe pain, restricted upper extremity mobility and skin changes such as pallor in the affected limb. At the time of admission (Day 0), the patient was increasingly cardiopulmonary unstable and required norepinephrine support preoperatively.

### Radiological examination

While at the local hospital prior to transfer to our medical university center (Day -1), computed tomography (CT) images of the left upper extremity were obtained using a contrast agent. The images revealed extensive subcutaneous edema, consistent with a soft tissue infection. There was no evidence of a local abscess or gas formation along the fascial planes. Furthermore, no signs of osteomyelitis were observed (Fig. 1c).

### Laboratory examination

Laboratory examinations on admission (Day 0) revealed a white blood cell (WBC) count markedly elevated at 15.34 × 10^9^/L and elevated C-reactive protein (CRP) levels at 299 mg/L. Additionally, procalcitonin (PCT) levels were elevated at 101.72 µg/L, confirming the presence of a severe bacterial infection. The patient also presented with an acute renal failure indicated by a creatinine level of 6.21 mg/dL and a urea level of 268.7 mg/dL, along with a glomerular filtration rate (GFR) of 7 mL/min. Thrombocytopenia, reflected by platelet counts as low as 27 × 10^9^/L, was indicative of a DIC. Liver function tests showed elevated liver enzymes, further indicating early liver failure. Overall, these laboratory investigations revealed significant findings indicative of systemic infection and multiorgan failure.

### Diagnosis

On admission (Day 0), the patient was diagnosed with a severe soft tissue infection of the left upper extremity, complicated by compartment syndrome and multiorgan failure, including acute renal failure, early liver failure, and DIC.

### Treatment and clinical course

**Fig. 2:**
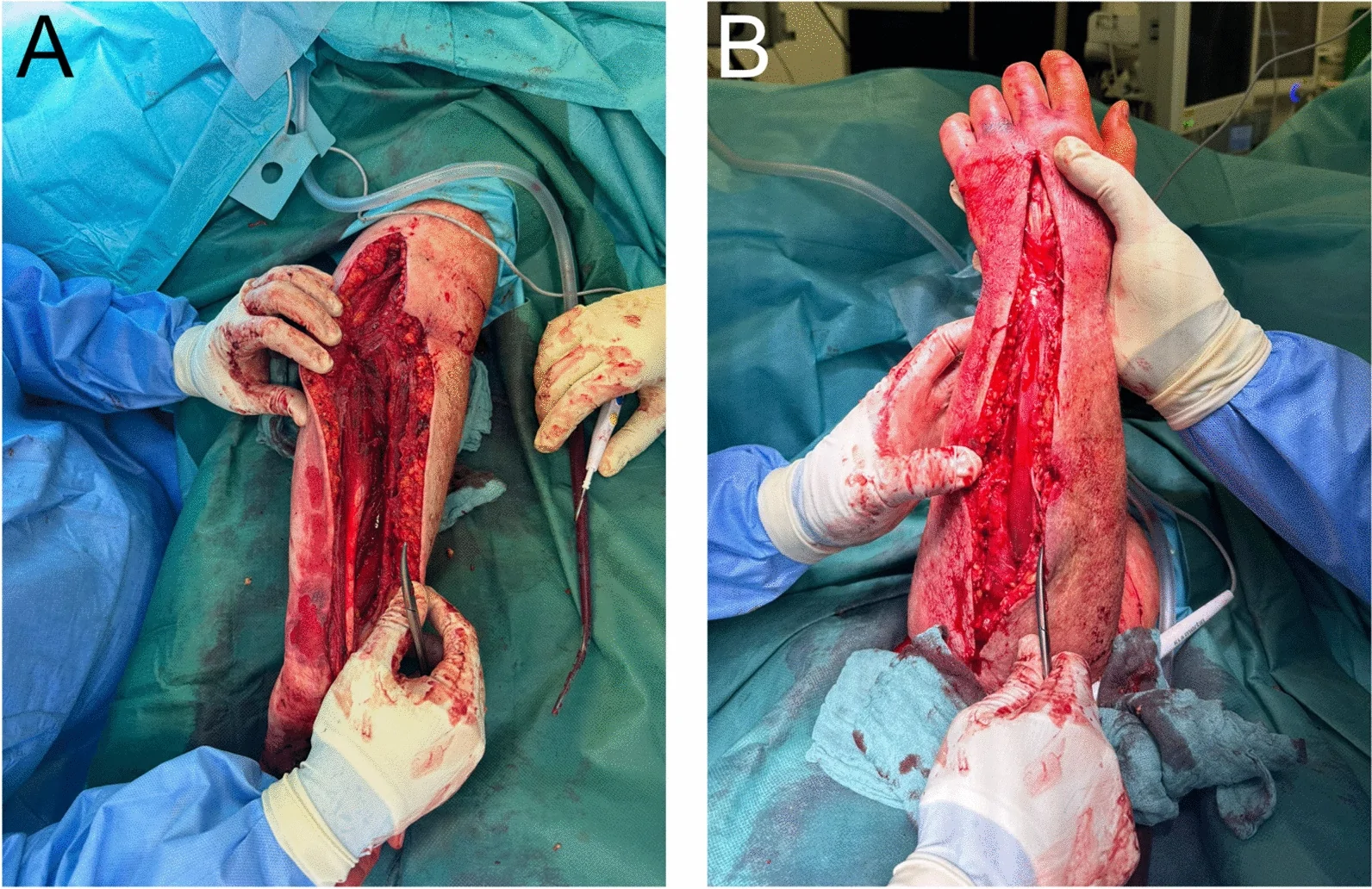
Intraoperative findings during the first surgical intervention. **a** Surgical exploration revealed extensive involvement of the subcutaneous tissue and musculature on the ventral side of the forearm. **b** The dorsal forearm was also incised, corresponding to the site of the initial scratch injury

Upon admission to the emergency room (ER, Day 0), the antibiotic therapy with Meropenem initiated by the referring local hospital was continued. Blood cultures were obtained (Day 0) but showed no detectable pathogens. Due to the critically low platelet count mentioned earlier, the patient received three units of platelets preoperatively to manage her thrombocytopenia. After examination and stabilization in the ER, the patient was immediately taken to the operating room (OR) for operative intervention (Day 0). A fasciotomy of both the superficial and deep anterior (Fig. 2a) as well as the posterior compartments (Fig. 2b) was performed. The musculature appeared initially vital. To release pressure from the median nerve, the carpal tunnel was released. There was a noticeable tightness in the area near the elbow, prompting the decision to extend the incision further proximally. No signs of necrotizing fasciitis were observed, but diffuse purulence was noted at depth. After extensive irrigation with Lavanox^®^ (Serag-Wiessner, Naila, Germany), a polyhexanide-based wound irrigation solution with antimicrobial properties, a moistened abdominal pack was placed ventrally in the forearm, while the dorsal wound was temporarily covered with a synthetic polytetrafluoroethylene and polyurethane containing skin substitute (EpiGARD®, Biovision, Ilmenau, Germany).

Following surgery (Day 0), the patient was transferred to the intensive care unit (ICU) for close monitoring and management.

**Fig. 3:**
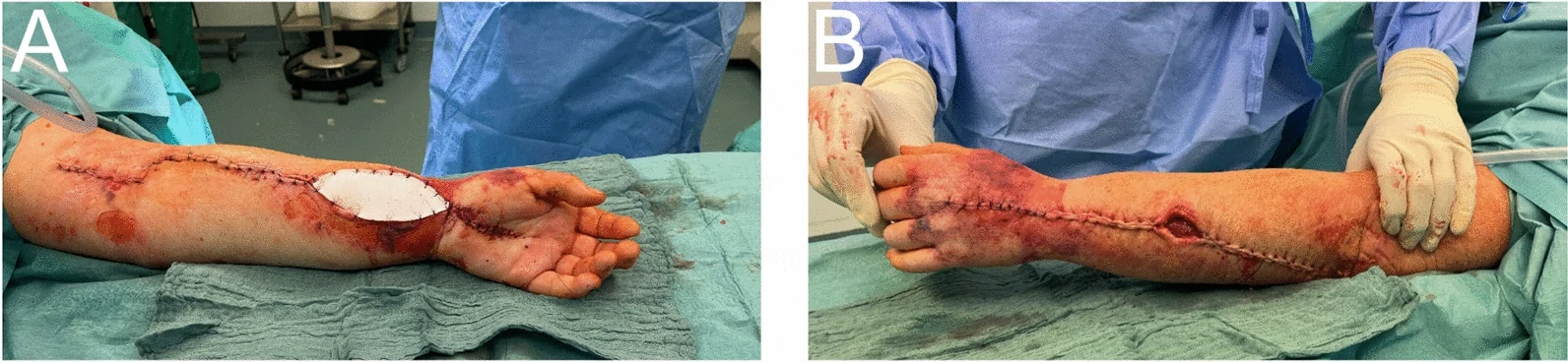
Wound closure following the second surgical intervention. **a** An EpiGARD^®^ wound covering was applied to the ventral forearm, where tissue tension prevented wound edge approximation. **b** On the dorsal side, the area requiring wound coverage was significantly smaller, allowing for partial closure

The next day (Day 1), a second-look surgery was performed. Examination of the musculature revealed only a few localized areas of necrosis measuring a few millimeters, along with intermittent small coagula. All muscle tissues appeared vital. A non-adherent silicone-coated nylon contact layer (Mepitel^®^, Mölnlycke, Düsseldorf, Germany) was applied to the area of the flexor tendons, and the wound was covered with EpiGARD^®^ (approximately 5 × 8 cm) (Fig. 3a). The dorsal side of the hand could also be closed. However, a complete skin closure in the area of the muscle compartment and the tendons in the forearm was not possible (Fig. 3b). Mepitel® was again applied in the area of the tendons, followed by closure of the remaining wound with EpiGARD^®^.

Cultures obtained from the necrotic tissue during the initial surgery (Day 0) and the second-look operation (Day 1) identified *Streptococcus pyogenes* (Group A), while only one culture grew *Staphylococcus aureus*, which was considered a contamination. Following availability of microbiological results, antibiotic therapy was adjusted according to the antibiogram to Ampicillin/Sulbactam, which was effective against both *Streptococcus pyogenes* and the potentially contaminated isolate of *Staphylococcus aureus*.

On admission (Day 0), the patient presented with acute renal failure characterized by anuria and rising kidney values. Arterial blood gas analysis (ABG) revealed metabolic acidosis, indicated by a pH value of 7.2. Diuresis gradually improved following an aggressive fluid substitution; however, renal parameters continued to deteriorate, necessitating hemodialysis (Day 2). Over time, kidney values began to improve, and dialysis was discontinued.

**Fig. 4:**
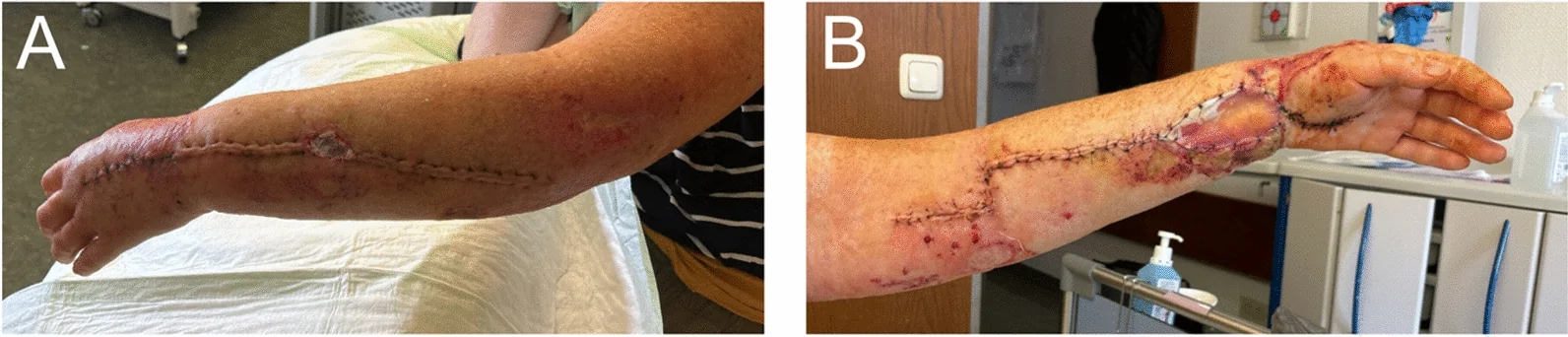
Postoperative condition of surgical wounds 10 days after the initial intervention. **a** Postoperative assessment revealed persistent swelling of the left upper limb. **b** Necrotic and discolored areas were observed in the distal forearm

After 3 days (Day 3), the patient was transferred from the ICU to a regular ward. In total, eight surgical procedures were performed, including the initial fasciotomy (Day 0), the second-look operation (Day 1), and further revision procedures (Day 3, 8, 15, 19, 24, 32; Fig. 4a, b). Initial revision surgeries did not yield any bacterial growth from intraoperative microbiological samples. However, during the sixth surgical procedure (Day 19), *Pseudomonas aeruginosa* was identified on both the dorsal and ventral sides. Antibiotic therapy was consecutively escalated to intravenous Piperacillin/Tazobactam. Several samples returned positive for *Pseudomonas aeruginosa* during the sixth revision as well, while one sample came back positive for *Streptococcus mitis/Streptococcus oralis* and *Veillonella rogosae* on the dorsal side. These findings were interpreted as a contamination of microbiological samples. Already existing antibiotic therapy covered the abovementioned potentially contaminated isolates.

The seventh surgical procedure (Day 24) indicated a significant decline in the healing process, with putrid secretion noted in the wound area on the dorsal side, in contact with the olecranon bursa. As a result, the initial incision was extended over the olecranon, followed by bursectomy of the olecranon bursa. The flexor carpi radialis tendon was found to be necrotic, resembling overcooked asparagus, and was deemed non-functional, resulting in its resection.

**Fig. 5:**
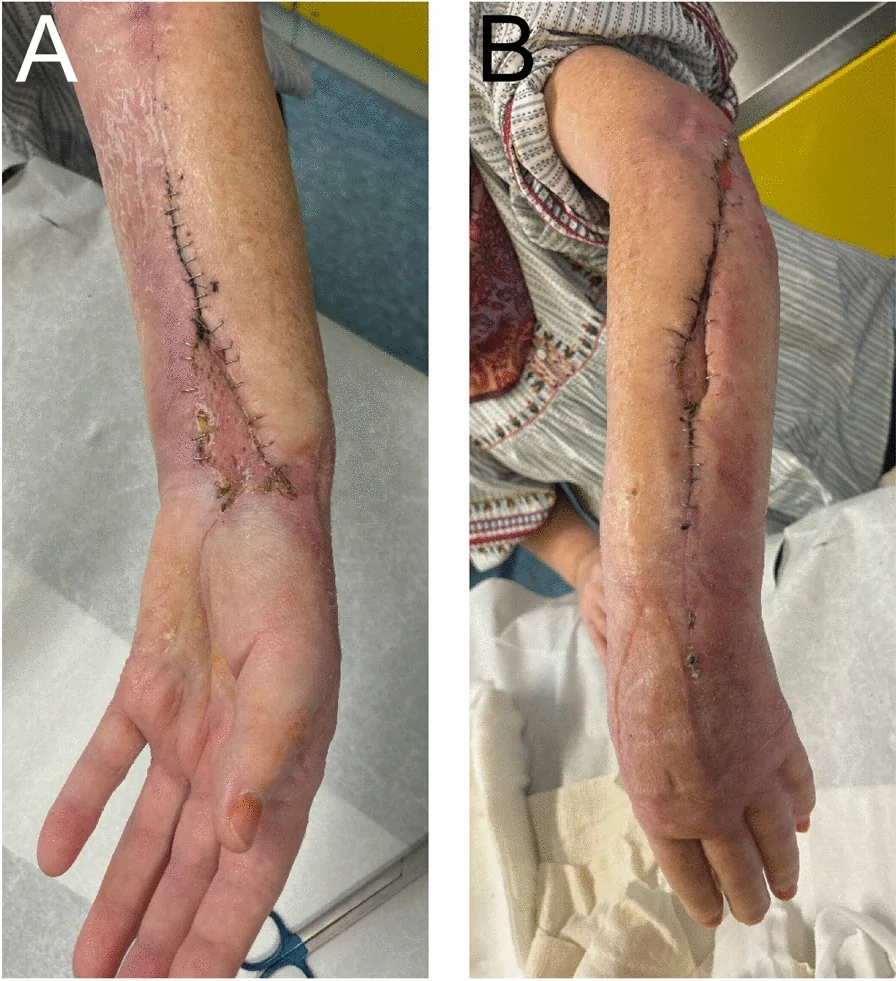
Wound condition following split-thickness skin grafting. **a** Postoperative evaluation demonstrated successful integration of the grafts on the ventral forearm. **b** Split-thickness skin grafts were also applied to the dorsal forearm

During the last surgical intervention (Day 32), the wound bed appeared uninflamed with healthy granulation tissue. A secondary wound closure was performed. In areas where this was not possible, split-thickness skin grafting was performed anteriorly (Fig. 5a) and posteriorly (Fig. 5b) in the area of the forearm and elbow. After 6 weeks, the patient was discharged (Day 44).

During follow-up, 59 days after discharge (Day 103), the split-thickness skin graft demonstrated good integration and healing, indicating successful surgical management of the initial condition. However, despite adequate treatment, including regular physiotherapeutic interventions during the hospital stay, the patient exhibited significant functional deficits, including severely restricted elbow movement with an extension/flexion range of 0–10–70°, limited shoulder external and internal rotation of 45–0–45°, stiffened fingers with minimal active mobility, an almost immobile wrist, and the inability to achieve a full fist closure. To address these limitations, the patient underwent intensive rehabilitation immediately after hospital discharge, including physiotherapy and occupational therapy interventions. The patient reported experiencing mild symptom relief and noticeable improvements in finger mobility after each session. Despite these efforts, functional deficits remain (wrist extension/flexion: 10–0–20°; forearm pronation/supination: 80–0–80°), highlighting the profound impact of the initial condition and its treatment.

A chronological overview of the patient’s clinical course, diagnostic workup, surgical interventions, and follow-up is provided in Table 1.

**Table 1:** Timeline of clinical presentation, diagnostic workup, surgical interventions, and follow-up in a patient with severe upper extremity soft tissue infection complicated by compartment syndrome

Time point	Clinical event
Day -14	Scratch injury to the left forearm caused by a student
Day -14 to Day -1	Conservative management with antibiotic therapy; progressive pain, swelling, blistering, and fever
Day -1	Admission to local hospital; initiation of intravenous Ampicillin/Sulbactam and Clindamycin; CT imaging performed
Day 0	Clinical deterioration; escalation of antibiotic therapy to Meropenem; transfer to tertiary referral center
Day 0	Diagnosis of severe SSTI with compartment syndrome, sepsis, acute renal failure, DIC, and early liver failure
Day 0	Emergency fasciotomy, carpal tunnel release, debridement, and ICU admission
Day 1	Second look operation
Day 2	Initiation of hemodialysis
Day 3	Transfer from ICU to regular ward; first revision procedure
Day 8	Second revision procedure
Day 15	Third revision procedure
Day 19	Fourth revision procedure; detection of *Pseudomonas aeruginosa* and escalation of antibiotic therapy to Piperacillin/Tazobactam
Day 24	Fifth revision procedure; bursectomy and resection of necrotic flexor carpi radialis tendon
Day 32	Final surgical procedure with secondary wound closure and split-thickness skin grafting
Day 44	Hospital discharge and initiation of intensive rehabilitation
Day 103	Follow up examination (59 days after discharge); good graft integration but persistent functional deficits

## Discussion

SSTIs encompass a wide range of conditions, from relatively benign infections to severe, life-threatening complications. Understanding the spectrum of SSTIs is critical for appropriate diagnosis and management. This case report describes the severe progression of an SSTI in the upper extremity in a patient who had recently recovered from COVID-19. Despite its seemingly benign onset, the infection rapidly progressed and required prompt and aggressive multidisciplinary management. The distinction between limited phlegmon, often presenting as cellulitis, and severe phlegmon can sometimes be ambiguous, making it challenging for clinicians to determine the point at which an infection transitions from a localized, superficial condition to a more serious, systemic infection.

Limited phlegmon is typically characterized by an infection involving the dermis and subcutaneous tissues. Commonly associated with *Staphylococcus aureus* and *Streptococcus pyogenes*, this type of infection often arises in the context of skin breaches, such as cuts, insect bites, or surgical wounds, or, as in this case, a scratch injury. Clinically, it may present with local infection signs such as erythema, swelling, and warmth around the affected area, with localized pain that may or may not be accompanied by systemic symptoms. Treatment for limited phlegmon generally includes appropriate antibiotic therapy, immobilization and local wound care.

The transition to severe phlegmon, however, can be subtle and is not always straightforward [5]. Severe phlegmon is characterized as a progressive and invasive infection, often accompanied by extensive purulence and necrosis formation. In some cases, such as the one described here, progression can be fulminant and tissue inflammation can lead to an increase in interstitial pressure, leading to an impending or manifest compartment syndrome. In this stage, the infection extends beyond the superficial layers of the skin and fat to involve deeper structures, including fascia and muscle. Patients may exhibit significant systemic responses, such as fever, tachycardia, and hypotension, indicating a systemic inflammatory response. The presence of these systemic signs often necessitates immediate and aggressive surgical intervention to prevent further complications, including necrotizing fasciitis, sepsis, and septic shock, which can result in organ failure and potentially be fatal. In extreme cases, such as rapidly progressing or treatment-resistant infections, limb amputation may be required to preserve the patient’s life. This necessity for aggressive surgical intervention often results in functional deficits, underscoring the critical nature and profound consequences of this condition. Recognizing the factors that influence this transition is critical for improving patient outcomes [6]. Factors such as the patient’s underlying health status, immune response, and the virulence of the infecting organism can all play a role in the severity of the infection. In patients with comorbidities, such as diabetes or immunosuppression, the likelihood of developing a deeper SSTI, such as severe phlegmon, is increased [7]. Moreover, the delay in seeking medical attention can also exacerbate the condition, highlighting the importance of early recognition and treatment of SSTIs [8]. The present case is noteworthy because a seemingly minor scratch injury progressed to a life-threatening upper extremity soft tissue infection with compartment syndrome and multiorgan failure, ultimately requiring eight surgical procedures before successful limb salvage could be achieved.

The case underscores the complexity of managing severe SSTIs. The initiation of empirical broad-spectrum antibiotic therapy with Meropenem was warranted due to the severity of the infection, which progressed to sepsis, necessitating coverage for resistant strains. Subsequent adjustment to Ampicillin/Sulbactam based on intraoperative microbiological cultures reflects best practices in antibiotic stewardship. However, the emergence of nosocomial resistant organisms such as *Pseudomonas aeruginosa* during the course of treatment illustrates the challenges of maintaining optimal antimicrobial coverage without fostering resistance.

Despite successfully managing the infection, the patient’s prolonged immobilization and the extent of tissue damage resulted in significant functional deficits, including limited elbow and wrist mobility and stiffened fingers. This emphasizes the long-term impact of severe SSTIs, even when managed appropriately. The patient’s intensive rehabilitation program illustrates the importance of addressing physical impairments to improve quality of life, though functional recovery may remain incomplete.

## Conclusion

This case demonstrates how a seemingly minor scratch injury can progress to a severe soft tissue infection with compartment syndrome, sepsis, and multiorgan failure. Early recognition of clinical deterioration, prompt surgical intervention, appropriate antimicrobial therapy, and close multidisciplinary collaboration were essential for successful infection control and limb preservation. Clinicians should remain vigilant when evaluating patients with rapidly progressive soft tissue infections, as timely diagnosis and aggressive treatment may prevent life-threatening complications and improve outcomes.

## Data Availability

All data generated or analyzed during this study are included in this published article. No additional datasets were generated or analyzed.
